# High-Throughput Analysis of Plasma Hybrid Markers for Early Detection of Cancers

**DOI:** 10.3390/proteomes2010001

**Published:** 2014-01-13

**Authors:** Jung-hyun Rho, Paul D. Lampe

**Affiliations:** Translational Research Program, Human Biology and Public Health Sciences Divisions, Fred Hutchinson Cancer Research Center, 1100 Fairview Avenue North, Seattle, WA 98109, USA; E-Mail: jrho@fhcrc.org

**Keywords:** cancer, early detection, biomarker, plasma, autoantibody, immune complex, glycan

## Abstract

Biomarkers for the early detection of cancer in the general population have to perform with high sensitivity and specificity in order to prevent the costs associated with over-diagnosis. There are only a few current tissue or blood markers that are recommended for generalized cancer screening. Despite the recognition that combinations of multiple biomarkers will likely improve their utility, biomarker panels are usually limited to a single class of molecules. Tissues and body fluids including plasma and serum contain not only proteins, DNA and microRNAs that are differentially expressed in cancers but further cancer specific information might be gleaned by comparing different classes of biomolecules. For example, the level of a certain microRNA might be related to the level of a particular protein in a cancer specific manner. Proteins might have cancer-specific post-translational modifications (e.g., phosphorylation or glycosylation) or lead to the generation of autoantibodies. Most currently approved biomarkers are glycoproteins. Autoantibodies can be produced as a host’s early surveillance response to cancer-specific proteins in pre-symptomatic and pre-diagnostic stages of cancer. Thus, measurement of the level of a protein, the level of its glycosylation or phosphorylation and whether autoantibodies are produced to it can yield multi-dimensional information on each protein. We consider specific proteins that show consistent cancer-specific changes in two or three of these measurements to be “hybrid markers”. We hypothesize these markers will suffer less variation between different individuals since one component can act to “standardize” the other measurement. As a proof of principle, a 180 plasma sample set consisting of 120 cases (60 colon cancers and 60 adenomas) and 60 controls were analyzed using our high-density antibody array for changes in their protein, IgG-complex and sialyl-Lewis A (SLeA) modified proteins. At *p* < 0.05, expression changes in 1,070 proteins, 49 IgG-complexes (11 present in the protein list) and 488 Lewis X-modified proteins (57 on the protein list) were observed. The biomarkers significant on both lists are potential hybrid markers. Thus, plasma hybrid markers have the potential to create a new class of early detection markers of cancers.

## 1. Introduction

Cancer is a common cause of death in the world, and the burden is increasing for many reasons such as population aging, environmental exposures, cancer-associated lifestyles (e.g., diet, obesity smoking and physical activity) and late diagnosis with low survival rates [1]. Early detection of cancer is known to improve the five-year survival rate and reduce treatment costs. However, current screening for cancer relies on imaging methods, biopsy pathology and a few classical biomarkers that are not suitable for widespread population screening. Biomarkers present in serum or plasma fulfill several requirements for an effective widespread screening test. The samples are obtainable by non-invasive means and could be integrated with regular check-ups to select individuals that need to undergo more follow-up diagnostic tests. They are generally acceptable to people, and the assays utilized are usually low cost. The major cancer killers such as lung, colon and breast cancer are the most viable initial targets of such tests either as first line screens or to guide decision making from indeterminate imaging tests. For example, advanced low-dose helical computed tomography can detect cancerous lung nodules but with a high false positive rate. A recent large study shows that only 4% of participants with positive helical computer tomography (CT) screening results were finally diagnosed with lung cancer [2]. Falsely detected patients need to undergo additional diagnostic procedures, some of which carry medical risks along with unnecessary anxiety [3,4]. For colon cancer, the current gold standard detection method is colonoscopy, which allows removal of polyps and biopsy of cancer at the same time with high specificity (95%) for large polyps. However, colonoscopy is not a simple, inexpensive or generally acceptable test applicable to the entire at-risk population. Although there are some prescreening tests such as stool assays, they generally have lower patient compliance and miss many cancers [5]. Therefore, more and better blood biomarkers are needed.

At this point there are only approximately 20 protein/glycoprotein biomarkers that are FDA approved [6] (see Table 1). Despite new “omic” technologies used for discovery, few new markers have been adopted in the clinic [7] due to multiple scientific and regulatory factors. Systematic reasons for this are multi-fold: Many studies suffer from design flaws that lead to false discovery including the use of convenience samples without well-matched controls, the use samples taken at surgery with the inherent problem that disease symptoms can cause large changes in stress/inflammatory markers, and discovery and validation studies are often underpowered [8]. Furthermore, people are different genetically and have different environmental exposures that affect their blood proteome independent of disease. Tumor generated biomarkers can also be dependent on tumor size. Related to this, a recent mathematical modeling study predicted that a single ovarian cancer cell would require 8–10 years of growth to 25 mm diameter in order to shed sufficient CA125 into the blood stream to be detectable [9]. Finally, translation of putative markers discovered with sophisticated technologies into clinically useful assays that can then be validated in large clinical sample sets is often difficult and always time consuming. 

**Table 1:** A list of protein and glycoprotein cancer biomarkers in clinic.

Protein, glycoprotein and glycan markers	Cancer type	Source	Clinical use	Known glycosylations
Alpha-fetoprotein (AFP)	Liver	Blood	Staging	Sialydated [10]
Beta-human chorionic gonadotropin (Beta-hCG)	Choriocarcinoma	Urine/blood	Staging, prognosis, treatment response	N- and O-glycans [11]
CA125 (MUC16)	Ovarian	Blood	Monitoring	High mannose, complex bisecting N-glycans.Type 1 and 2 O-glycans [12]
CA15-3 (MUC1)	Breast	Blood	Monitoring	TF antigen (core 1) and sialyated [13]
CA19-9	Pancreas	Blood	Monitoring	Sialyl Lewis A [14]
CEA (carcinoembryonic antigen)	Colon	Blood	Monitoring	Lewis X and Y, high mannose N-glycan [13]
Chromogranin A (CgA)	Neuroendocrine tumors	Tumor	Diagnosis, prognosis	O-glycan [15]
EGFR	Non-small cell lung cancer	Tumor	Treatment selection	N- and O-glycans [16], sialylated and fucosylated [17]
Epididymis protein 4 (HE4)	Ovarian	Blood	Monitoring	N-glycan [18]
Fibrin/Fibrinogen (gamma chain)	Bladder	Urine	Monitoring	N-glycan and fucosylated [19]
HER2/neu	Breast, gastric, esophageal	Tumor	Monitoring, prognosis and treatment selection	N-glycan [20]
KIT	GI stromal tumor and mucosal melanomas	Tumor	Diagnosis, treatment selection	N-glycan [21]
Prostate-specific antigen (PSA)	Prostate	Blood	Screening and monitoring	Single N-glycan [22], sialylated [23]
Thyroglobulin	Thyroid	Tumor	Monitoring	N-glycan [24]

A variety of approaches is being taken to overcome these difficulties. The goal of this review is to provoke thought about research strategies that combine different types of biomarkers in a manner that might reduce the impact of some of these issues. Our presentation is not meant to be comprehensive nor is it meant to be a general review of technologies or strategies for early detection. 

## 2. A New Paradigm for Novel Blood-Based Cancer Markers: Panels of Hybrid Markers

To improve early detection of cancers, blood-based biomarkers have a minimal benchmark that they must perform better by themselves or in combination with current ones [e.g., cancer antigen 125 (CA125); cancer antigen 19-9 (CA19-9), carcinoembryonic antigen (CEA); and prostate-specific antigen (PSA)]. Both efforts in biomarker discovery and the study of the genetics of tumors have shown that cancers are heterogeneous diseases by genomic and histological analyses, and single markers may not accurately diagnose all tumor types. For example, Stage I epithelial ovarian cancers are divided into four different histological subtypes (serous, mucinous, endometrioid and clear cell carcinomas) correlating with distinct genetic patterns [25]. Breast cancers are heterogeneous by cancer subtype (ductal and lobular) [26], histological grade [27], estrogen (ER)/progesterone (PR) receptor status, ERBB2 amplification [28], and patient age [29]. Other common cancers including lung cancer also display extensive heterogeneity associated with multiple biological factors [30]. Heterogeneity is further complicated by genetic changes at advanced stages and after response to therapy. 

To successfully detect these heterogeneous cancers, combinations of new biomarker candidates with current markers are being tested for improved sensitivity and specificity. Many initial attempts along these lines have been reported but these will require considerable follow-up in multiple sample sets before they could be considered for use in the clinic. For example, in ovarian cancer, elevation of OVX1 antigen in blood has been found to be complementary to CA125, and detection of either marker has achieved a sensitivity of 80% [31]. CA125 correlated with low CEA can discriminate primary ovarian cancer from colon cancer [32]. When CA19-9 was combined with haptoglobin and SAA, the sensitivity and specificity for the detection of pancreatic adenocarcinoma was increased to 81.3% and 95.5% from 77.3% and 91.1%, respectively [33]. In colon cancer, a combination of six serum markers including CEA, CYFRA21-1, ferritin, osteopontin (OPN), seprase and anti-p53 autoantibody showed a comparable sensitivity to fecal immunochemical testing with 68.9% versus 72.7% at 98% specificity [34]. An optimal panel is not necessarily made of the top individual markers from one class of biomolecules. For example, the performance of markers from more than one type of measurement (e.g., protein level) might be complemented by the levels of cancer specific glycosylation, phosphorylation, mRNA or microRNAs. Furthermore, if there was a biological relationship between the markers (e.g., the level of a protein and its extent of phosphorylation), the ratio of the levels might improve panel performance. For instance, a specific phosphorylation event on a protein could be consistently 10-fold higher in cancer samples but the protein level might vary 20-fold between people so just examining protein phosphorylation levels would potentially yield a wide spread of overlapping values. Taking the phosphorylation and protein level of that sample into account (e.g., a ratio) would yield a more consistent, better performing “hybrid” biomarker.

## 3. Serum and Plasma as a Source of Biomarkers

Plasma and serum samples are rich sources of potential biomarkers that can be studied using various strategies. They include but are not limited to genomic, proteomic, glycomic and metabolomic markers. Circulating free DNAs [35], methylated tumor-specific DNAs [36] and microRNAs [37] are examples of potential genetic markers that hold promise through multiplex and next generation sequencing advances. However, due to the variable presence of DNases and RNases in blood, we believe tumor tissue will remain the main area of focus for genetic material for post diagnosis treatment purposes. For example, non-small-cell lung cancers (NSCLC) harboring gain-of-function mutations in the epidermal growth factor receptor (EGFR) gene or anaplastic lymphoma kinase (ALK) genes are now likely to be treated with first-line tyrosine kinase inhibitors [38]. 

Related markers such as mRNA paired with protein expression, protein expression paired with phosphorylation levels, or microRNAs paired with protein and/or mRNA expression are all examples of potential “hybrid” marker candidates. In order to use data to illustrate some of the key points, we will focus our further discussion on proteins, glycoproteins and autoantibodies, areas of which we have some expertise. However, we want to emphasize that we do not mean to suggest the approaches we use are better or preferred over other approaches and/or classes of biomarkers. 

Protein biomarkers are probably the most intensely researched and clinically practiced class of blood biomarkers. Most currently used cancer biomarkers (~20) are proteins [6]. However, even with the recent advances in high-dimensional proteomic methodologies, the rate of approval of new FDA-approved protein tests has declined [39]. In addition to the regulatory environment, one potential reason for fewer new tests might be the conventional paradigm of looking only for changes in one class of biomolecule. Since people are genetically diverse, have very different environmental exposures and have different underlying disease status, the level of any protein alone can vary significantly independent of disease. Searching for modified proteins as biomarkers for this purpose is attractive for many reasons. Proteins are reflective of ongoing cellular physiology and modifications from alternative splicing, and post-translational modifications such as phosphorylation and glycosylation have been shown to occur in a cancer-specific manner. 

### 3.1. Plasma and Serum Proteomes

**Figure 1:**
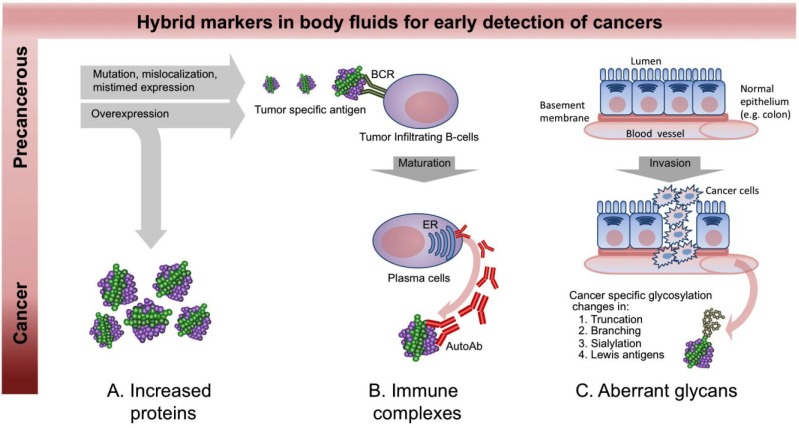
The presence of “hybrid markers” in blood. (**A**) Proteins secreted by stromal cells or lysed tumor cells can reflect disease states. Measurements of increased (or decreased) expression can be used for early detection, diagnosis, prognosis, risk stratification and therapeutic monitoring of cancer; (**B**) Autoantibodies, are by definition associated with autoimmune diseases, but also may form during cancer progression. Self-proteins with up-regulated expression, point mutation or altered post-translational modifications could become autoantigenic. These antigens can activate tumor infiltrating B cells to mature and produce autoantibodies. This immune surveillance often occurs early during a disease process. In blood, these autoantibodies can be complexed with their antigens as immune complexes or unbound if the autoantigen is not present; (**C**) Glycoprotein expression and the type and extent of glycosylation on specific proteins are known to be altered in a tumor-specific manner by loss or excessive expression of certain glycan biosynthetic pathways. In addition, invasive tumors disrupt the normal architecture of polarized epithelial cells resulting in mislocalization of soluble glycoproteins including mucins.

The human blood proteome is complex with more than tens of thousands of different proteins depending on how protein modifications are counted (Figure 1A). The quantitative dynamic range of plasma proteins is estimated to span 10–12 orders of magnitude as exemplified by the difference between albumin (35–50 × 10^9^ pg/mL) and interleukin 6 (0–5 pg/mL). In addition to classical plasma proteins synthesized in the liver, blood contains proteins that are secreted or leaked from other tissues [39]. The only commonly used cancer-screening marker is prostate-specific antigen (PSA), which belongs to the kallikrein-related peptidase family. PSA is present at a very low level in healthy young male populations and gradually increases with age. A total PSA serum level greater than 4 ng/mL usually leads to follow-up biopsies of the prostate to look for evidence of cancer [40]. The performance of PSA testing in population screening is controversial due the fact that 20%–50% organ-confined cancer patients have less than 4 ng/mL of total PSA and many that have higher levels of PSA might not result in aggressive disease that kills the individual [41]. Several other cancer biomarkers present in blood are FDA approved for limited purposes. CA125 (MUC16) can be used for diagnostic and recurrence purposes for ovarian cancer [42]. Similarly, carcinoembryonic antigen (CEA) is a marker approved under limited circumstances to monitor colon cancer recurrence but other markers that might complement CEA performance or serve independently for early detection of adenomas and invasive colon cancer are still only under development [43]. Additional protein markers mainly used in the clinic to guide treatment and prognosis include alpha-fetoprotein (AFP), beta-2-microglobulin (B2M), beta-human chorionic gonadotropin (beta-hCG), CA15-3/CA27.29, CA-125 and HE4 [6]. 

### 3.2. Autoantibody Markers

Human blood contains abundant immunoglobulins including autoantibodies specific to self-molecules even in healthy people [44]. Antibodies to self-molecules may be generated because proteins are over/aberrantly expressed, are mislocalized, have point mutations [45], have altered post-translational modifications (PTMs) [46], are misfolded, are truncated due to aberrant splicing or proteolytic cleavage [47] or they may mimic pathogenic antigens [48]. The host immune system may also respond to a class of self-molecules modified during cell apoptosis [49], proteins with certain physicochemical properties [50,51], and proteins selectively expressed in tumors [52]. Tumor-infiltrating lymphocytes (TILs) are recruited to tumors at early stages. Among different TILs, tumor-infiltrating B cells (TIB) (e.g., CD20^+^ B cells) may take up tumor antigens through the B cell receptor and produce autoantibodies (autoAbs) (Figure 1B). An *in vivo* experiment demonstrated that severe combined immune-deficient mice had detectable human immunoglobulin in their serum after subcutaneous transplantation of human lung cancer tissue [53]. The post-engraftment production of antibodies suggests that the human lung cancer tissue contained TIB. The development of autoantibodies by autoreactive B cells has multiple clinical implications. Coexistence of tumor infiltrating T and B cells has been shown to be associated with higher survival rates and lower relapse rates in breast [54], ovarian [55], cervical [56], colon [57] and lung cancers [58,59]. Stimulatory cytokine and chemokine treatments that enhance tumor infiltrating T cell (e.g., CD4^+^ and CD8^+^ T cells) responses can affect outcome [60]. 

Autoantibodies have been proposed to be excellent biomarker candidates since their levels can be amplified as part of an early immune response so that even low levels of tumor antigen could lead to a robust signal. Additionally, antibodies are high-affinity, structurally stable proteins that can be easily quantified by a variety of detection methods that can be readily translated into clinical settings. In a study of lung cancer, IgG autoantibodies to p53 were present in 30% of patients at the time of diagnosis, and they were specific to a p53 missense mutation event that occurs early in 60%–70% of people with this cancer [61]. A combined IgG autoantibody panel of MUC1-STn, MUC1-Core3 and p53 detected 30% of colon cancers at prediagnostic stages [62]. However, these studies detected only the presence of autoantibody and ignored the potential added value of the autoantigen. 

### 3.3. Carbohydrate Markers

Many of the current clinical cancer biomarkers are particular carbohydrate structures (e.g., CA19-9) or are glycoproteins (e.g., CA125, CA15-3 and CEA, a more comprehensive list shown in Table 1). Approximately 50% of all proteins are estimated to be glycosylated [63] and glycan abundance and their micro- and macro-heterogeneity can be changed in a disease-specific manner (Figure 1C). Reported cancer specific N-glycan changes are observed as increased β1-6 branching via enhanced expression of GlcNAc transferease V, increased sialylation attached to outer Galβ1-4GlcNAc units, and increased sialyl Lewis A and X structures (both are selectin ligands) [64]. For O-glycan type modifications, incomplete glycosylation and truncated O-glycans result in Tn antigen (GalNAc-α1-O-Ser/Thr), T antigens (Thomsen-Friedenreich, Galβ1-3GalNAc-α1-O-Ser/Thr) and sialyl-Tn (sialyl2-6αGalNAc-a1-O-Ser/Thr). As for N-glycan modifications, O-glycans show increased sialic acid and sialyl Lewis A and X structures [65]. Glycosylations beyond these N- and O-types also show cancer-specific changes. Glycosphingolipids were found to be increased in some cancers including Burkitt’s lymphoma, melanoma and neuroblastoma. Hyaluronan is a repeating disaccharide of (GlcAβ1–3GlcNAcβ1–4)_n_ found associated with tumor stroma mostly as a free polymer. Cancers can also show decreased sulfated proteoglycan expression including dermatan sulfate, keratin sulfate and heparan sulfate [64]. 

Carbohydrate-related biomarkers can have independent diagnostic value as well as supplemental benefit to known markers for better specificity and sensitivity [66,67,68]. Multiple methods have been used to investigate glycoproteomes. Glycoprotein markers can be identified by mass spectrometry after immunoprecipitation or lectin affinity capture with carbohydrate structure analysis derived from their masses [69,70,71]. Antibody microarrays have also been used for carbohydrate analysis by capturing glycoproteins followed by detection of modifications by lectins or carbohydrate-specific antibodies [72,73,74]. One study used 58 different antibodies to mucins, matrix proteins, adhesion proteins, and cytokines on an array to capture potential CA19-9 antigen carrying proteins from sera of pancreas cancer patients. They found that the presence of CA19-9 on MUC5AC or MUC16 showed improved sensitivity over the standard CA19-9 assay alone [75].

Our recent report utilized a high dimensional antibody array to discern how broad certain cancer specific carbohydrate modifications are across a significant portion of the plasma proteome. Glycoproteins in blood or tissue samples were specifically captured by over 3,000 antibodies on an array, and the glycan moieties on proteins were detected by two different fluorescently labeled anti-carbohydrate-specific antibodies (sialyl Lewis A and Lewis X) [76]. The utility of the platform is discussed below further in a context of hybrid marker discovery.

## 4. Enhanced Performance of Hybrid Markers: A Potential Future Direction of Early Detection Biomarker Discovery Research

Only very few studies have examined the performance of “hybrid” markers and those usually involve examining modifications of known glycoproteomic markers such as PSA, CEA, CA-125 and mucins. As a test of the hybrid marker concept using a broad screening approach, we examined a small plasma sample set of late stage CRCs where we measured protein, IgG-autoantibody-antigen complex and Lewis-X modified protein relative levels by antibody microarray. The sample set contained 30 plasma samples from CRC patients (6 IIIa, 10 IIIb, 5 IIIc and 9 IV stages) [76]. 

**Figure 2:**
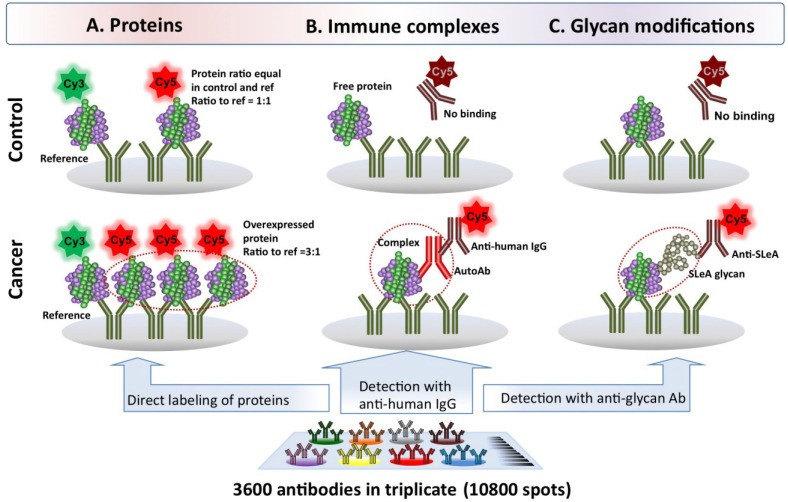
Antibody microarray methods to profile proteins, autoantibody-antigen complexes and glycan modifications. Our antibody arrays contain ~3,200 distinct antibodies printed in triplicate along with control spots. (**A**) The proteins in the sample are quantified on the array after removal of IgG and albumin followed by direct labeling with Cy5 and incubation on the array with a Cy3 labeled reference sample as an internal control; (**B**) The same antibody array platform has been successfully implemented to detect autoantibody-antigen complexes and (**C**) glycoproteins modified with a specific glycan of interest. Essentially, the array performs affinity fractionation to purify the proteins to the ~3,200 specific spots on the array. Then, the bound proteins are probed with either a fluorescently labeled antibody specific to human IgG for autoantibody-antigen complex detection or to the cancer modified carbohydrate such as sialyl Lewis A (the CA19-9 antigen).

Our antibody array platform is capable of measuring thousands of analytes in a high-throughput manner (Figure 2) [44,76,77,78]. Given the high affinity and specificity of antibody binding, these assays do not require extensive sample preparation such as pre-fractionation to reduce the complexity of plasma proteome. Our antibody arrays currently contain ~3,200 antibodies printed in triplicate (10,800 total spots/slide), and a schematic of the proteomic method of profiling is presented in Figure 2A. Briefly, IgG and albumin are depleted from the plasma samples, then the proteins are tagged with Cy5 (case or control) or Cy3 dyes (reference sample) followed by co-incubation on array. Case and control sample concentration ratios to the reference are then used for comparison [44,77]. The antibody microarray platform allows us to perform not only proteomic analysis but also to determine whether the proteins bound to the array are complexed with human autoantibodies (*i.e*., autoantibody-antigen complexes) or have cancer-specific glycosylation modifications, as illustrated in Figure 2B,C. In essence, the array fractionates and purifies the proteins to localize them to the specific spots on the array. We can then probe the bound proteins with either a fluorescently labeled antibody specific to human IgG for autoantibody-antigen complex detection or antibody specific for the cancer modified carbohydrate such as Lewis X. The combination of our high density antibody array with detection of autoantibody-antigen complexes and glycomic modifications in a discovery setting have each recently been reported as technological advances [44,76].

**Figure 3:**
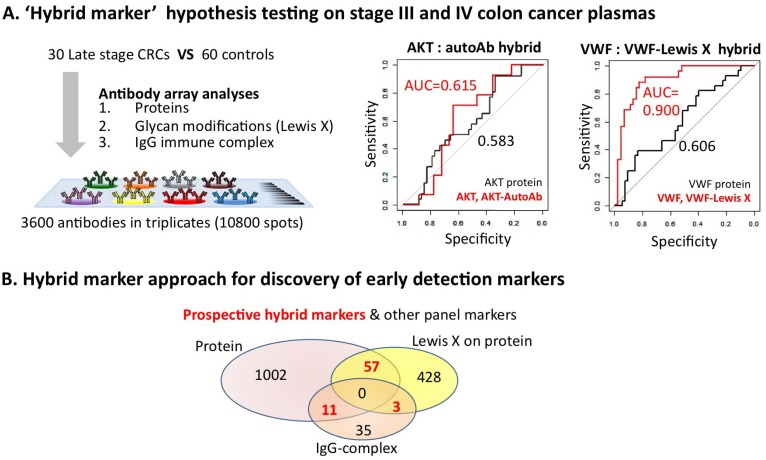
Hybrid marker’s improved biomarker performance over protein expression alone. (**A**) Plasma samples from colon cancer patients were analyzed by antibody array for their relative protein levels, IgG-autoantibody-antigen complexes and Lewis X modifications. Hybrid markers consisting of the protein component and cancer specific glycosylation (here Lewis X) or in a complex with IgG-immune complex showed enhanced biomarker utility as measured by AUC over the protein performance by itself; (**B**) A global view of the abundance and performance of hybrid markers was performed by comparing plasma from 60 patients with adenomas and 60 patients with colon cancer to 60 controls using each of the platforms. The Venn diagram shows the total number of biomarker candidates statistically significantly (*p* < 0.05) identified with each profiling technique and the overlap of protein identity between the three classes (potential hybrid markers). Whether hybrid markers perform well enough alone or are good candidates to be part of a panel of biomarkers for colon cancer early detection, diagnosis or prognosis remains to be determined.

In support of the “hybrid marker” hypothesis, here we describe how the combination of all three categories (protein, Lewis X modified protein and IgG-complex) of biomarkers could indicate potential hybrid biomarkers that have improved performance. We compared the same 30 late stage colorectal cancer samples and the 60 healthy controls as discussed above. We found many protein biomarkers with moderate AUC values perform better when autoantibody or glycosylation data for that marker was included. For example, the AUC for Akt protein increased from 0.58 to 0.615 when its presence in an autoantibody-antigen complex was added (Figure 3A). Von Willebrand factor protein alone only has an AUC of 0.61 but it is increased to 0.90 when Lewis X-modifications are considered [76]. This demonstrates that broad screening for hybrid markers may (1) lead to a discovery of novel biomarkers with high combined performance, as well as (2) improve the performance of protein markers that alone have little clinical utility to the point that they may be useful.

To get a better overall view of whether hybrid markers might be useful, we tested plasma samples from 60 adenoma patients (30 larger adenomas and 30 early adenomas) and 60 CRC patients (11 I, 17 IIa and 2 IIb, 6 IIIa, 10 IIIb, 5 IIIc and 9 IV stages) using proteomic, IgG-complex and Lewis X detection. The proteomic analysis revealed that 1,070 proteins were differentially expressed in the adenoma and cancer samples with statistical significance (*p* < 0.05). The IgG-complex analysis detected 49 differentially expressed complexes in the adenoma and CRC patients at *p* < 0.05. Of the 49, 11 showed statistically significant differences in protein expression. Detection of Lewis X modifications found 488 potential markers (*p* < 0.05), 57 of which showed significant protein changes and three of which were autoantigens as demonstrated by the presence of complexes with IgG (Figure 3B). Thus, this analysis found 71 examples of potential hybrid markers that will need to be tested in further sample sets to determine their ultimate utility. 

## 5. Conclusions

Although “omic” technologies have revolutionized biomarker research, few discoveries using these techniques have made it into the clinic. Investigators that are trying to discover biomarkers typically utilize specific methods that are targeted to one biomarker class. In this article, we show that combinations of markers from different biomolecule classes may lead to detection of “hybrid biomarkers” that are not only differentially expressed at the protein level but that also have different cancer specific modifications. Specifically, we define a potential ‘hybrid marker’ as a molecule that shows statistically significant differences by two distinct measures, and the combined performance may allow tests with better sensitivity and specificity. Blood contains many different classes of measurable molecules, and in this article, three characteristics of one protein are considered as sources of hybrid markers: protein concentration, cancer specific glycosylation, and immune complex formation. The idea is that one aspect of a biomarker can be made into a stronger classifier by characterizing other measurable characteristics of the marker. 

The concept of hybrid markers is not new to the biomarker research community. Complexes and modifications of known markers have been studied for improved performance for some time albeit usually in a targeted manner. For example, improved performance has been shown for PSA measured in the free form, total form (complexes of PSA and other proteins) and, even in some cases as IgM-immune complexes for prostate cancer detection [79,80]. However, discovery efforts that examine thousands of potential candidates over multiple platforms are more unique. The advancement of “omic” research now has the potential to allow one or several groups to perform multiple screening analyses as we demonstrated with our high-density antibody arrays. By this approach, classifiers with only moderate performance characteristics in the one dimension might show dramatic improvement as a hybrid marker (e.g., VWF is Figure 3A). 

We suppose that application of the hybrid approach to biomaker discovery in blood would depend on the type of markers being combined. For autoantibody-antigen pairs, cancers in organ sites with high vascularity and blood flow might be most suitable. For example, lung tissue has a rich blood supply (13.60 mL/min/g) compared to the brain (0.43 mL/min/g) [81]. Different organs show various levels of protein secretion and protein glycosylation. The Lewis X structure mentioned here is preferentially expressed in specific cancers including colon [82], breast [83] and pancreas [84]. Therefore, the choice of the classes of biomarkers to be combined for hybrid markers might be at least partially dictated by the cancer being studied. 
